# Mapping Gene-by-Gene Single-Nucleotide Variation in 8,535 Mycobacterium tuberculosis Genomes: a Resource To Support Potential Vaccine and Drug Development

**DOI:** 10.1128/mSphere.01224-20

**Published:** 2021-03-10

**Authors:** Danai Papakonstantinou, Steven J. Dunn, Simon J. Draper, Adam F. Cunningham, Matthew K. O’Shea, Alan McNally

**Affiliations:** a Institute of Microbiology and Infection, College of Medical and Dental Sciences, University of Birmingham, Birmingham, United Kingdom; b Jenner Institute, University of Oxford, Oxford, United Kingdom; c Institute of Immunology and Immunotherapy, MRC Centre of Immune Regulation, University of Birmingham, Birmingham, United Kingdom; Albert Einstein College of Medicine

**Keywords:** *Mycobacterium tuberculosis*, tuberculosis, TB, single nucleotide polymorphisms, SNPs, drug targets, vaccine candidates, single-nucleotide variation, tuberculosis vaccines

## Abstract

Tuberculosis (TB) is responsible for millions of deaths annually. More effective vaccines and new antituberculous drugs are essential to control the disease. Numerous genomic studies have advanced our knowledge about M. tuberculosis drug resistance, population structure, and transmission patterns. At the same time, reverse vaccinology and drug discovery pipelines have identified potential immunogenic vaccine candidates or drug targets. However, a better understanding of the sequence variation of all the M. tuberculosis genes on a large scale could aid in the identification of new vaccine and drug targets. Achieving this was the focus of the current study. Genome sequence data were obtained from online public sources covering seven M. tuberculosis lineages. A total of 8,535 genome sequences were mapped against *M. tuberculosis* H37Rv reference genome, in order to identify single nucleotide polymorphisms (SNPs). The results of the initial mapping were further processed, and a frequency distribution of nucleotide variants within genes was identified and further analyzed. The majority of genomic positions in the M. tuberculosis H37Rv genome were conserved. Genes with the highest level of conservation were often associated with stress responses and maintenance of redox balance. Conversely, genes with high levels of nucleotide variation were often associated with drug resistance. We have provided a high-resolution analysis of the single-nucleotide variation of all M. tuberculosis genes across seven lineages as a resource to support future drug and vaccine development. We have identified a number of highly conserved genes, important in M. tuberculosis biology, that could potentially be used as targets for novel vaccine candidates and antituberculous medications.

**IMPORTANCE** Tuberculosis is an infectious disease caused by the bacterium Mycobacterium tuberculosis. In the first half of the 20th century, the discovery of the Mycobacterium bovis BCG vaccine and antituberculous drugs heralded a new era in the control of TB. However, combating TB has proven challenging, especially with the emergence of HIV and drug resistance. A major hindrance in TB control is the lack of an effective vaccine, as the efficacy of BCG is geographically variable and provides little protection against pulmonary disease in high-risk groups. Our research is significant because it provides a resource to support future drug and vaccine development. We have achieved this by developing a better understanding of the nucleotide variation of all of the M. tuberculosis genes on a large scale and by identifying highly conserved genes that could potentially be used as targets for novel vaccine candidates and antituberculous medications.

## INTRODUCTION

Mycobacterium tuberculosis is the causative agent of tuberculosis (TB), an ancient disease that remains one of the biggest causes of infectious disease mortality worldwide (1). According to the WHO, TB accounts for the death of over 1.3 million people every year, while almost 25% of the world’s population is latently infected (1). Limitations of diagnostic methods and the emergence of multidrug-resistant (MDR) and extensively drug-resistant (XDR) TB further compound the global health challenge (2).

A major obstacle in combating TB is the lack of an effective vaccine (1, 3). The efficacy of Mycobacterium bovis bacillus Calmette-Guérin (BCG), the only commercially available TB vaccine to date, is geographically variable and provides little protection against pulmonary disease (1, 3). At the same time, large-scale genomic analyses have shown that although the M. tuberculosis genome exhibits relatively low levels of sequence variation, and undergoes no obvious homologous recombination, strain diversity is more extensive than previously thought (2). The two human M. tuberculosis complex species (M. tuberculosis *sensu stricto* and Mycobacterium africanum) are composed of seven distinct, globally distributed lineages (L1 to L7), with an eighth lineage recently reported (2, 4).

The spread of M. tuberculosis in eight human-adapted lineages, in combination with the complex host immune response to the organism, makes the development of a new globally applicable vaccine or drug more challenging (5). Sequence variation within a drug target-binding site could lead to reduced binding affinity and drug resistance (6, 7). Similarly, gene variation is an important consideration in understanding the likely value of a target antigen in a vaccine, since gene variation is largely responsible for protein variability in the bacterial population (8). In the field of TB, an ideal vaccine would have universal application and an efficacy of >50% against adult pulmonary TB (9). The traditional vaccinology approach has focused on developing TB vaccines based on immunodominant antigens (5). With this approach, only one candidate targeting adults with latent disease has shown protective efficacy of >50%, in phase IIb clinical trials, despite decades of research (5, 10).

Advances in whole-genome sequencing have broadened our knowledge of M. tuberculosis in areas such as M. tuberculosis population structure (2), transmission patterns, and outbreaks (11) and the evolution of drug resistance (2, 6, 12). At the same time, using reverse vaccinology has led to the identification of potential immunogenic M. tuberculosis antigens and T-cell epitopes (13). However, we still do not know what constitutes protective immunity in TB (3). In addition, a large number of M. tuberculosis genes and proteins are underexplored for their potential as targets for drug and vaccine strategies. If we are to identify new vaccine and drug targets, then it is important to first identify genes that are highly conserved across the entire species. As such, a better understanding of the sequence variation of every M. tuberculosis gene across all lineages could be beneficial for the identification of new proteins and may have potential application in novel drug and vaccine discovery.

We have conducted a large-scale genomic study to provide a high-resolution analysis of the single-nucleotide variation of every M. tuberculosis gene across 8,535 genomes. We have combined our analysis with functional annotation from multiple sources. We show that there are extremely high levels of variation in genes known to be involved in drug resistance. Conversely, our approach also shows extreme conservation across the M. tuberculosis population in genes involved in latent TB infection and stress responses. These genes are conserved across seven lineages, which suggests an essential role in TB biology, and may constitute novel targets for TB drug and vaccine development.

## RESULTS

### Collation of a data set encompassing the known sequenced genomic diversity of M. tuberculosis.

Our final analysis included 8,535 genome sequences from key genomic study data sets (see Table S1 in the supplemental material). The raw genome data from these studies were mapped against the H37Rv reference genome (14). All seven global lineages were represented in our data, with L1 to L4 being predominant, which is in keeping with sequenced data previously published (12). The breakdown of the lineages in our data was as follows: L4, 56.8%; L2, 21.4%; L3, 12.8%; L1, 7.7%; and L5, L6, and L7, 1.3%. The population structure in our data set is representative of the known sequenced strain diversity of M. tuberculosis (2) (Fig. S1).

10.1128/mSphere.01224-20.1 FIG S1 Core SNP phylogeny of 8,535 M. tuberculosis genomes. A core SNP alignment of 8,535 genome sequences was created using the snippy-core function in Snippy and the program snp-sites (v2.4.0). All the PE-PPE regions were masked. An algorithm with rapid bootstrapping (100 bootstrap replicates) was used to reconstruct an SNP phylogeny, using a general GTR+CAT approximation algorithm. One *M. canettii* genome was used as an outgroup (brown dashed lines). The known sequenced global M. tuberculosis diversity was demonstrated (L1 to L7). L2 to L4 (modern lineages) arise from a common branch, whereas L1 and L5 to L7 have a separate diverging common ancestor (ancient lineages). Coloring of the lineages is as follows: L1, pink; L2, blue; L3, purple; L4, red; L5, green; L6, dark orange; L7, yellow. Download FIG S1, PDF file, 2.8 MB. Copyright © 2021 Papakonstantinou et al. 2021 Papakonstantinou et al. https://creativecommons.org/licenses/by/4.0/ This content is distributed under the terms of the Creative Commons Attribution 4.0 International license.

10.1128/mSphere.01224-20.4 TABLE S1 Data sets included in the analysis. Data sets, published online, were retrieved from the European Nucleotide Archive (https://www.ebi.ac.uk/ena). Our combined data set included genomic sequences from all over the world in order to achieve adequate representation (of sequenced online data) of all the human M. tuberculosis lineages. Project accession numbers are available for reproduction. Download TABLE S1, PDF file, 0.2 MB. Copyright © 2021 Papakonstantinou et al. 2021 Papakonstantinou et al. https://creativecommons.org/licenses/by/4.0/ This content is distributed under the terms of the Creative Commons Attribution 4.0 International license.

### Nucleotide variation is distributed across the genome within the M. tuberculosis population, with a number of variant hot spots.

**FIG 1:**
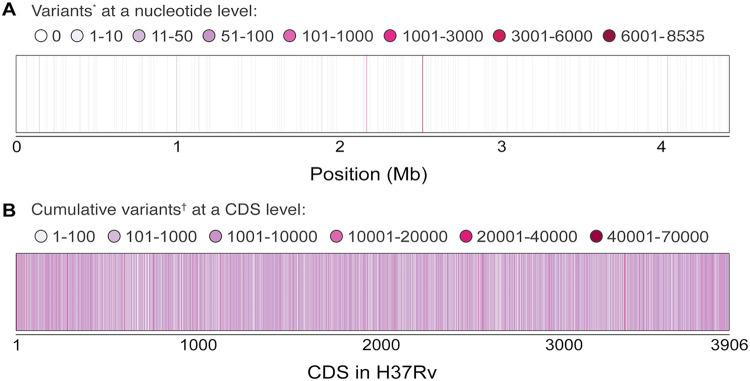
Distribution of the sequence variation at a genomic position and CDS level. (A) Variants*, number of genomes with a variant across H37Rv. Mapping of 8,535 genomes against H37Rv demonstrated that 92.2% of H37Rv genomic positions were conserved upon comparison. A small number of positions contained a large amount of genomes containing a variant (1 to 10 genomes had variants in 7.13% of genomic positions of H37Rv, 10 to 100 genomes had variants in 0.49% of H37Rv genomic positions, and 100 to 8,530 genomes had variants in 0.16% of H37Rv genomic positions. (B) Cumulative variants†, cumulative number of variants across the genomes at a CDS level. CDS from 1 to 3906 and their total number of variants across the data set. At a CDS level, all coding sequences have some degree of variation. Mobile elements, repeat regions, transposases, and RNAs were excluded from this analysis.

Our analysis revealed that the majority of genomic positions in the M. tuberculosis H37Rv genome were completely conserved (92.2%) and that a small number of genomic positions contained variants across sequenced genomes in comparison to that of H37Rv (7.13% of the genomic positions had variants in 1 to 10 genomes, 0.49% had variants in 10 to 100 genomes, and 0.16% had variants in 100 to 8,530 genomes) (Fig. 1A and Fig. S2). However, all identified coding sequences (CDS) in the H37Rv genome had some level of sequence variation across the genomes analyzed (Fig. 1B). Summary statistics of the single nucleotide polymorphism (SNP) distribution at a CDS level showed a mean of 1,403.1 genomes from 8,535 genomes containing a nucleotide variant at a given position in a CDS and a median of 498.5 genomes containing a variant at a given position per gene. To remove potential bias, the results were normalized by gene length, resulting in a mean level of variation of 1.43654 genomes containing a mutation/bp and a median of 0.55407 genomes containing a mutation/bp per CDS.

**FIG 2:**
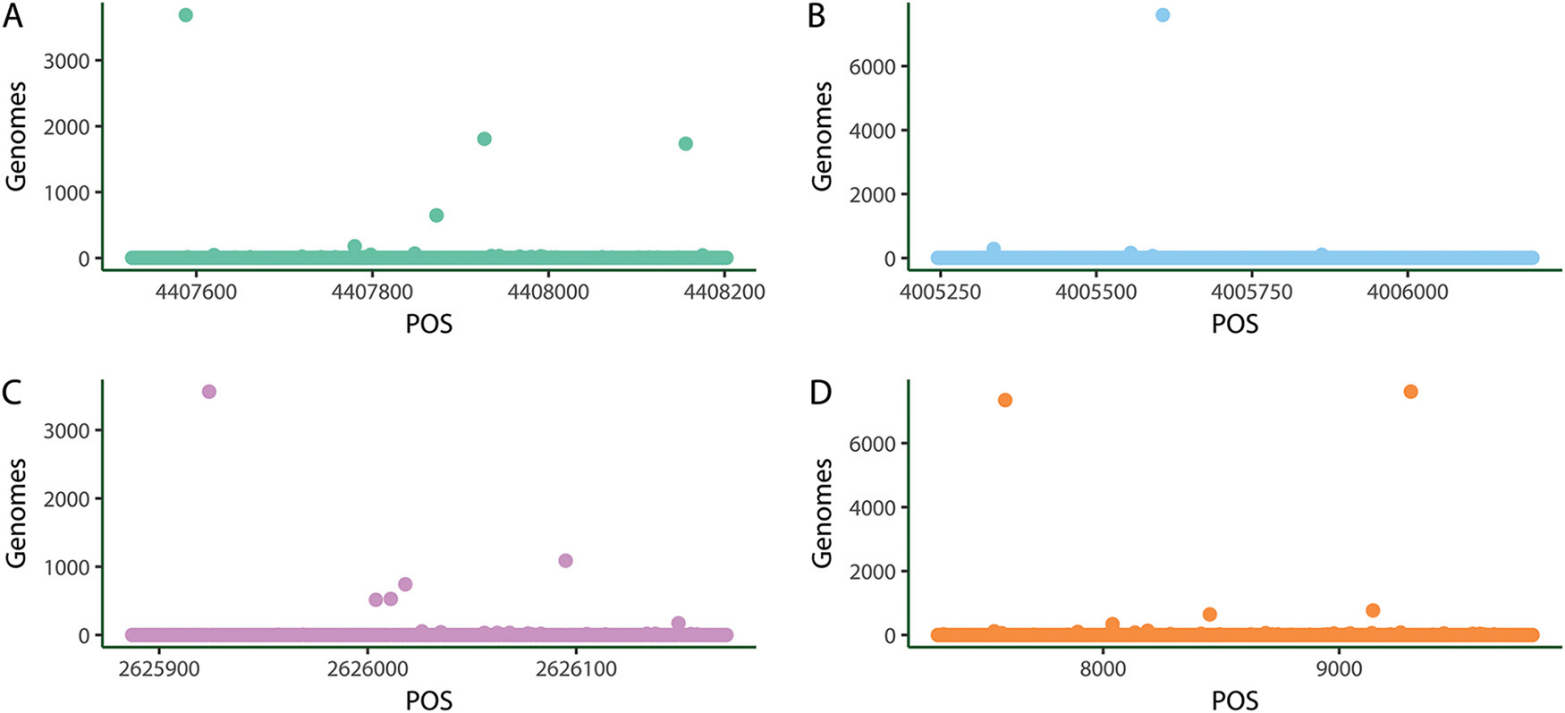
Variant hot spots within M. tuberculosis genes *gidB* (A), *fadE33* (B), *esxO* (C), and *gyrA* (D). Four representative examples of genes with high variation: *gidB*, a gene associated with streptomycin resistance (6); *fadE33* (a member of the *fadE* family), which plays a role in cholesterol metabolism (25); *esxO*, which belongs to the ESAT-6 group (16); and *gyrA*, associated with quinolone drug resistance (6). (A) *gidB.* The majority of genomic positions (POS) within *gidB* are conserved. However, 3,690 genomes had a variant at position 4407588, 1,811 genomes had a variant at position 4407927, and 1,734 genomes had a variant at position 4408156. (B) *fadE33.* The majority of genomic positions within *fadE33* are conserved. However, 7,603 genomes had a variant at position 4005607, and 279 genomes had a variant at position 4005335. (C) *esxO.* The majority of genomic positions within *esxO* are conserved. Examples of highly variable genomic areas within *esxO* are positions 2625924 and 2626095. Specifically, 3,561 genomes had a variant at position 2625924 and 1,088 genomes had a variant at position 2626095. (D) *gyrA. gyrA*, which is associated with drug resistance to quinolones (6), is 2,517 bp (bp) long, and across the analyzed population, the majority of genomic positions within *gyrA* are conserved. This gene exhibits variation only at certain positions, some of which are known to be associated with quinolone resistance (e.g., 7,345 genomes had a variant at position 7585). In addition, 7,609 and 769 genomes had a variant at genomic positions 9304 and 9143, respectively.

10.1128/mSphere.01224-20.2 FIG S2 Manhattan plot of the sequence variation of 8,535 genomes. The *x* axis represents the genomic positions of H37Rv, and the *y* axis represents the number of genomes in our data set having a variant. It is noted that the plot has a higher density closer to zero, as the majority of the genomic positions are completely conserved (when 8,535 genomes are mapped against H37Rv). The identification of variant hot spots is not evident in this graph. Please refer to Fig. 2 in the main article. Download FIG S2, PDF file, 0.8 MB. Copyright © 2021 Papakonstantinou et al. 2021 Papakonstantinou et al. https://creativecommons.org/licenses/by/4.0/ This content is distributed under the terms of the Creative Commons Attribution 4.0 International license.

Genes with high numbers of variants above the mean/median, such as *gyrA*, had hot spots within the gene at which the majority of the mutations occurred. The *gyrA* gene, which is associated with drug resistance to quinolones (6, 15), is 2,517 bp long, and across the analyzed population, the majority of genomic positions within *gyrA* are conserved (87.3%). This gene exhibits variation only at certain positions, some of which are known to be associated with quinolone resistance (e.g., 7,345 genomes had a variant at position 7585). Examples of important families of genes exhibiting high variation (e.g., *gidB* [6], *esxO* [16], *fadE33* [17]), due to such hot spots, are shown in Fig. 2.

### Identification of genes with high and low levels of single-nucleotide variation across the data set.

**FIG 3:**
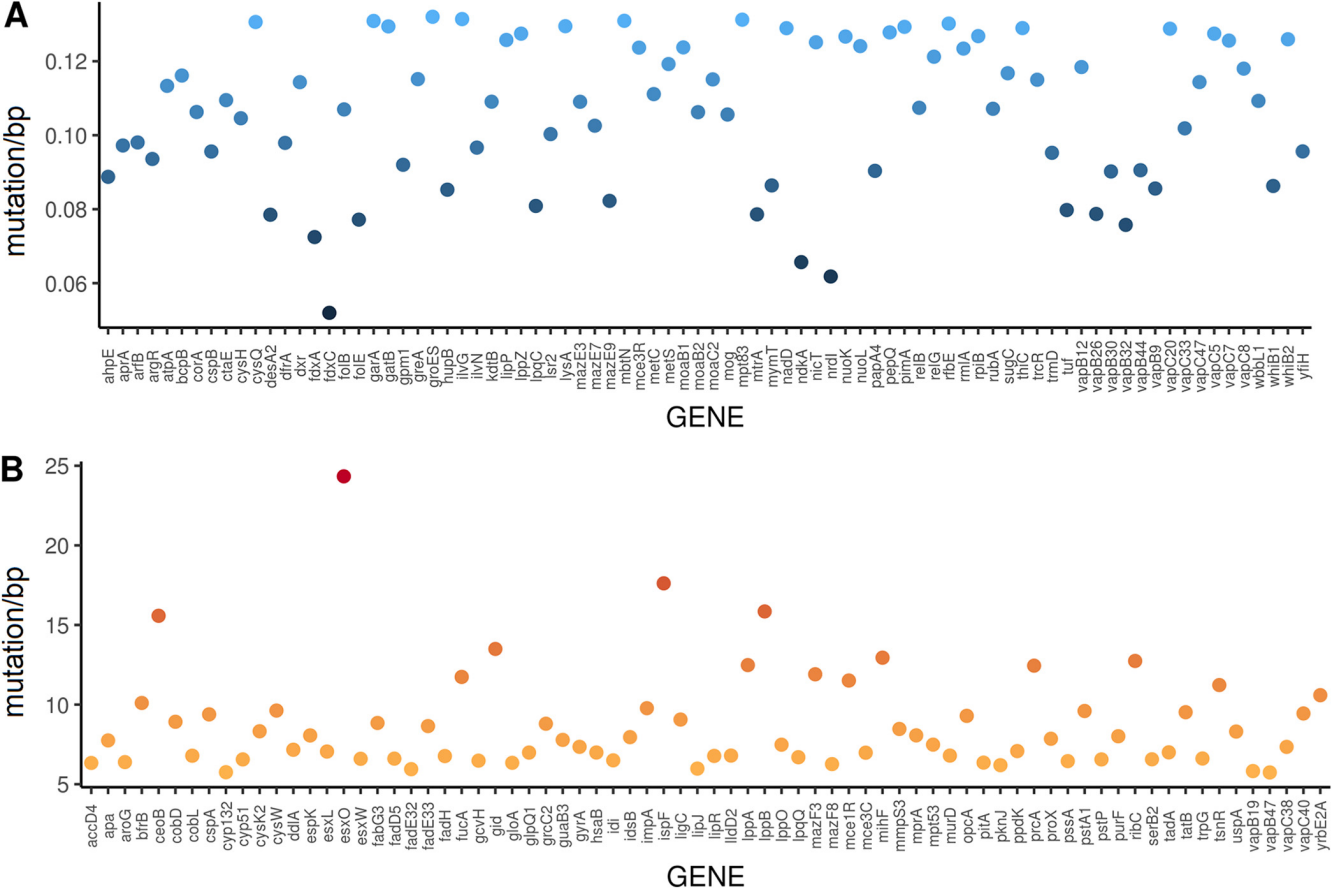
Distribution of the single-nucleotide variation of the genes present in the 5th (A) and 95th (B) percentiles. (A) Fifth percentile genes. Of the genes with functional annotation to H37Rv, the gene with the lowest number of variants across the analyzed genomes in the 5th percentile was *fdxC* (0.05 genomes containing mutations/bp), which encodes a ferredoxin. It was noted that toxin-antitoxin genes of group II are predominant in the 5th percentile. This graph does not contain genes that were not genetically characterized with reference to H37Rv (i.e., hypothetical proteins). Information on these genes can be found in Data Set S1 in the supplemental material. Please note that in order to remove potential bias, the results were normalized by gene length (mutations/bp). (B) Ninety-fifth percentile genes. The *esxO* gene is the first genetically characterized gene with the highest variation (24.3 genomes containing mutations/bp) across the analyzed genomes. Genes associated with drug resistance (e.g., *lppB*, *lppA*, *gidB)*, *fadD* and *fadE* families (e.g., *fadE33*, *fadE32*, *fadH*), and ESAT-6 genes are present in the 95th percentile. This graph does not contain genes that were not genetically characterized with reference to H37Rv (i.e., hypothetical proteins). Information on these genes can be found in Data Set S1 in the supplemental material. Please note that in order to remove potential bias, the results were normalized by gene length (mutations/bp).

We sought to identify genes that were highly conserved and those that contained high levels of sequence variation. We determined a statistical threshold for areas with higher and lower variability than normal. We identified genes in the 5th and 95th percentiles with respect to the amount of sequence variation present in all genomes analyzed (Fig. 3 and Data Set S1). When putative functions were assigned to genes (via clusters of orthologous protein groups [COGs]) in each of the percentiles, the majority of the proteins were uncharacterized or poorly characterized (Table S2). We used Mycobrowser (18) and UniProt (19) databases in an attempt to further characterize genes. The initial comparison of the broader COG categories (e.g., metabolism) did not show any statistically significant difference in gene function between the 5th and 95th percentiles (Table S3). After additional functional annotation using TubercuList categories (14), we noted that more genes related to cell wall and cell processes had high sequence variation (95th percentile, *χ*^2^ = 8.8108, *P* < 0.01) (Table S3). In addition, there were a number of functional groups of proteins that were significantly associated with one of the two percentiles, e.g., toxin-antitoxin (TA) in the 5th percentile and antimicrobial resistance genes in the 95th percentile (Table S3).

10.1128/mSphere.01224-20.5 TABLE S2 Functional annotation by TubercuList and COGs of the genes at the 5th and 95th percentiles. *^a^*Genes involved in cell wall and cell processes were predominant in the 95th percentile (*P* value = 0.002995) (TABLE S3). *^b^*Explanation of COG subcategories: [D] cell cycle control, cell division, chromosome partitioning, [M] cell wall/membrane/envelope biogenesis, [N] cell motility, [O] posttranslational modification, protein turnover, and chaperones, [T] signal transduction mechanisms, [U] intracellular trafficking, secretion, and vesicular transport, [V] defense mechanisms, [W] extracellular structures, [Y] nuclear structure, [Z] cytoskeleton, [A] RNA processing and modification, [B] chromatin structure and dynamics, [J] translation, ribosomal structure, and biogenesis, [K] transcription, [L] replication, recombination, and repair, [C] energy production and conversion, [E] amino acid transport and metabolism, [F] nucleotide transport and metabolism, [G] carbohydrate transport and metabolism, [H] coenzyme transport and metabolism, [I] lipid transport and metabolism, [P] inorganic ion transport and metabolism, [Q] secondary metabolite biosynthesis, transport, and catabolism, [R] general function prediction only, [S] function unknown; UC, unable to characterize by prediction tool. Download Table S2, PDF file, 0.2 MB. Copyright © 2021 Papakonstantinou et al. 2021 Papakonstantinou et al. https://creativecommons.org/licenses/by/4.0/ This content is distributed under the terms of the Creative Commons Attribution 4.0 International license.

10.1128/mSphere.01224-20.6 TABLE S3 Comparison of genes highlighted in the 5th and 95th percentiles. *^a^*The chi-square test with Yates’ continuity correction was performed for the comparison of functional protein groups between the 5th and 95th percentiles. We considered a *P* value of <0.05 as significant. *^b^*There was no statistical difference between the broader clusters of orthologous groups (COGs) of proteins (e.g., cellular processes and signaling) between the 5th and 95th percentiles. *^c^*It is noted that the number of genes related to the TubercuList functional category “cell and cell wall processes” was elevated in the 95th percentile (statistically significant difference). *^d^*Conversely, toxin-antitoxin (TA) family and genes induced under stress were more prominent (statistically significant difference) in the 5th percentile. *^e^*Statistically significant values are italicized. Download TABLE S3, PDF file, 0.02 MB. Copyright © 2021 Papakonstantinou et al. 2021 Papakonstantinou et al. https://creativecommons.org/licenses/by/4.0/ This content is distributed under the terms of the Creative Commons Attribution 4.0 International license.

10.1128/mSphere.01224-20.9 DATA SET S1 A full list of every M. tuberculosis gene identified in the 5th (i.e., low sequence variation genes) and 95th (i.e., high sequence variation genes) percentiles and its nucleotide variation. In this supplemental material, we have included genes genetically characterized with reference to H37Rv, as well as genes encoding hypothetical proteins. Download Data Set S1, XLSX file, 0.05 MB. Copyright © 2021 Papakonstantinou et al. 2021 Papakonstantinou et al. https://creativecommons.org/licenses/by/4.0/ This content is distributed under the terms of the Creative Commons Attribution 4.0 International license.

We quantified the number of synonymous and nonsynonymous mutations present in the high- and low-variation genes and the percentage of single-nucleotide substitutions for each. CDS in the 95th percentile have more genes with a higher percentage of nonsynonymous SNPs (median, 90.4% of substitutions are nonsynonymous) than genes in the 5th percentile (median, 53% of substitutions are nonsynonymous) (Fig. S3).

10.1128/mSphere.01224-20.3 FIG S3 Percentage of nonsynonymous SNPs at the 5th and 95th percentiles. (A) Ninety-fifth percentile genes associated with drug resistance (such as *gyrA, ceoB,* and *opcA*) are noted to be affected by more nonsynonymous SNPs. Genes in the 95th percentile have more genes with a higher percentage of nonsynonymous SNPs (median, 90.4% of substitutions are nonsynonymous) than genes in the 5th percentile (median, 53% of substitutions are nonsynonymous). (B) Fifth percentile genes. In the 5th percentile, we observed that one of the groups of genes affected by more nonsynonymous SNPs was the toxin-antitoxin (TA) genes. In comparison to the 95th percentile, fewer genes in the 5th percentile have nonsynonymous SNPs (median, 53% of substitutions are nonsynonymous). Download FIG S3, PDF file, 1.5 MB. Copyright © 2021 Papakonstantinou et al. 2021 Papakonstantinou et al. https://creativecommons.org/licenses/by/4.0/ This content is distributed under the terms of the Creative Commons Attribution 4.0 International license.

### Genes with high numbers of SNPs are associated with drug resistance and cell wall-associated processes.

Of the 186 genes in the 95th percentile displaying the highest levels of nucleotide variation, 72 contained a functional annotation in H37Rv (Table 1). The remaining 114 genes mainly encoded hypothetical proteins (Data Set S1). Only 14.5% of the genes in the 95th percentile were deemed essential in transposon mutagenesis studies (20).

**TABLE 1:** Protein prediction in the 95th percentile

Category^*a*^	COG(s)	Gene(s)	Protein information
Cellular processes and signaling [D],[M],[N],[O],[T],[U],[V],[W],[Y],[Z]^*b*^	D	*vapB47*	Antitoxin, TA group
	M	*gid* (6)	Associated with streptomycin resistance
	M	*murD*	Peptidoglycan biosynthesis
	O	*prcA* ^*c*^	Intermediary metabolism and respiration
	T	*pstP* ^*c*^	Regulatory proteins
	T	*mazF3*	Toxin, TA group
	U	*tatB*^*c*^ (35)	Probable transmembrane transporter
Information storage and processing [A],[B],[J],[K],[L]^*b*^	J	*tsnR, mihF*	Information pathways
	J	*tadA*	CMP-type deaminase domain protein
	K	*mce1R, mprA, pknJ*	Regulatory proteins
	K	*cspA*	Cold shock protein
	L	*gyrA* (6)	Associated with quinolone resistance
	L	*ligC*	DNA recombination and repair
Metabolism [C],[E],[F],[G],[H],[I],[P],[Q]^*b*^	C, H	*fadH,*c *idsB*	Lipid metabolism
	C, G, Q	*cyp132,*^*c*^ *cyp51,*^*c*^ *ppdK,*^*c*^*fucA, opcA*	Intermediary metabolism and respiration
	C, E	*aroG,*^*c*^ *glpQ1,*^*c*^*lldD2,*^*c*^ *mpt53, gcvH*	Intermediary metabolism and respiration
	E	*proX*	Transmembrane transporter activity
	E	*aroG, cysK2, trpG,*^*c*^ *serB2*^*c*^	Amino acid biosynthesis
	F	*purF*	Purine biosynthesis/purine salvage
	F, P	*ddlA,*^*c*^ *ceoB, uspA, pstA1, pitA*^*c*^	Cell wall and cell processes
	F, I, G, H	*guaB3,*^*c*^ *lipR, idi, impA*	Intermediate metabolism and respiration
	H	*ribC, cobD, cobL*	Riboflavin/cobalamin biosynthesis
	H, I	*accD4,*^*c*^ *fadD5, fadE32*	Involved in lipid metabolism
	H, I	*fadE33, pssA, fabG3*	Involved in lipid metabolism
	I, H	*lspF,*^*c*^ *grcC2, lipJ*	Intermediate metabolism and respiration
	P	*pstA1, cysW* ^*c*^	Transmembrane transporter activity
	P	*bfrB*	Iron storage protein
	Q	*mce3C* (25)	Virulence factor, Mce family
	Q	*yrbE2A*	Part of mce2 operon
Poorly characterized [R],[S]^*b*^	S	*apa*	Immunogenic, cell wall and cell processes
	S/UC	*hsaB*	Cholesterol catabolism
	S.UC	*vapC38, vapC40, vapB19, mazF8*	TA group
	S/UC	*lppA, lppB, lpqQ, lppO*	Possible lipoproteins
Unable to characterize (UC)	S/UC	*esxO, esxL, esxW*	ESAT-6-like protein
	S/UC	*espK*	ESX-1 secretion system
	S/UC	*mmpS3*	Determinant of intrinsic M. tuberculosis AMR

a Classification of clusters of orthologous protein groups (COGs) in the 95th percentile, combined with information from Mycobrowser (18) and UniProt (19). Additional information from the literature is individually cited within the table. Genes related to basic COG categories (e.g., metabolism) were observed in both percentiles. However, certain families, such as the *fadD* and *fadE* genes (e.g., *fadE33*, *fadD5*, *fadE32*), associated with fatty acid and cholesterol metabolism, were observed only in the 95th percentile (17). Genes related to pathogenesis of TB disease (e.g., ESAT-6/ESX genes) and antibiotic resistance (e.g., *gyrA*, *gidB*) are present. ESAT-6/ESX family genes were predicted as poorly characterized. The classification-involved proteins encoded by genetically characterized genes with reference to H37Rv. Protein prediction for the noncharacterized genes can be found in Data Set S1 in the supplemental material.

b COG subcategories are explained analytically in the legend to Table S2 in the supplemental material.

c A number of genes have been identified as high-confidence drug targets (22).

**FIG 4:**
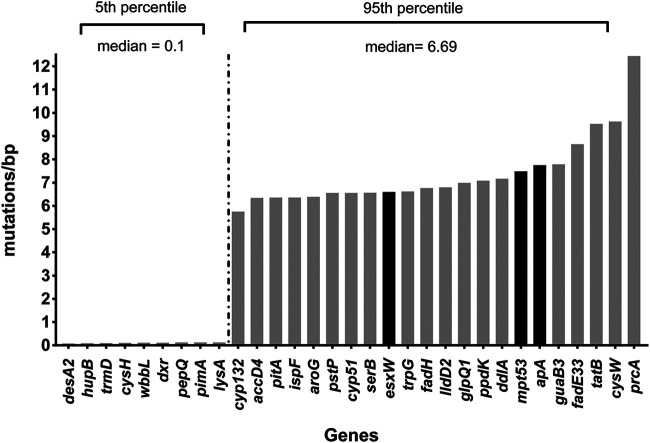
Drug and vaccine candidates previously proposed in the literature within the 5th and 95th percentiles. It is striking that a large number of genes demonstrating high single-nucleotide variation (95th percentile) in our data set have been previously proposed in the literature as desirable drug candidates (gray bars) (22). Few of these drug targets have been previously selected due to their location (e.g., *tatB*) or their biological function (e.g., *fadE33*) (17, 22). In addition, three genes in the 95th percentile have been previously proposed as potential vaccine candidates (*esxW*, *mpt53*, *apa*) (black bars) (5, 21). In fact, *esxW* encodes an immunogenic protein, which is present in a current subunit vaccine (ID93/GLA-SE) (5). A smaller number of genes that are highly conserved in our data set (5th percentile) have been previously proposed as drug targets (gray bars). Genes in the 5th percentile, previously proposed as drug targets, have a median ratio of 0.109272 mutations/bp. Genes in the 95th percentile, previously proposed as drug targets, have a median ratio of 6.691533 mutations/bp. Biological functions and functional annotation of the genes are described in Tables 1 and 2.

Functional groups of genes significantly overrepresented in the 95th percentile included drug resistance genes (e.g., *gyrA*) and others related to important M. tuberculosis families (e.g., ESAT-6), as well as cell wall and cell processes (e.g., *pitA*, *murD*). A large number of genes demonstrating high sequence variation were previously identified as vaccine (e.g., *esxW*, *apa*) (21) or drug (e.g., *accD4*, *fadE33*, *fadH*, *aroG*, *tatB*, *cyp132*) targets (22) (Fig. 4). Genes associated with drug resistance were also highly variable, including *gidB* (associated with low-level streptomycin resistance) (6), *gyrA* (quinolone-associated resistance gene) (6), and *ceoB* and *opcA* (observed in isoniazid-resistant strains) (23). We also found a high number of genomes with SNPs in *lppA* and *lppB*, which have been identified as novel targets for drug resistance to isoniazid, and in *lldD2*, a novel target for drug resistance to moxifloxacin (24). Genes from the ESAT-6/ESX family (e.g., *esxO*, *espK*), which are associated with virulence and disease pathogenesis (16), were present in the 95th percentile. In fact, *esxW* is considered highly immunogenic and is part of a current vaccine trial (5). Genes from other functional categories related to virulence were also identified (e.g., *tatB*, *mce*) (25). Only nine of the genes in the 95th percentile are associated with stress responses (e.g., *cspA*, *bfrB*) (Table S4). Finally, we report that several members of the PE-PPE family (e.g., PPE33, PE_PGRS47, PPE69, PPE18, PE_PGRS4, PPE59, PE_PGRS37, PPE19, PE_PGRS10, PE_PGRS9, PPE57) demonstrated high sequence variation in our initial analysis. However, due to the well-recognized problem of false-positive SNPs identified in these genes during mapping, we excluded them from our subsequent analysis, consistent with the approach of previous studies (26–28) (Data Set S1).

10.1128/mSphere.01224-20.7 TABLE S4 Ninety-fifth percentile high-variation genes previously found to be up- or downregulated under stress conditions. *^a^*Only nine of the genes in the 95th percentile are associated with stress responses (e.g. *cspA*, *bfrB*) (1–3), six of which were toxin-antitoxin (TA) genes*^b^* (4, 5). Download TABLE S4, PDF file, 0.1 MB. Copyright © 2021 Papakonstantinou et al. 2021 Papakonstantinou et al. https://creativecommons.org/licenses/by/4.0/ This content is distributed under the terms of the Creative Commons Attribution 4.0 International license.

### Highly conserved genes are associated mainly with stress responses and maintenance of redox balance.

Of a total of 186 genes occurring in the 5th percentile, 82 genes were functionally annotated in the H37Rv genome (14) (Table 2). The remaining 104 genes encoded mainly hypothetical proteins (Data Set S1). In contrast to the genes in the 95th percentile, 28% of the genes in the 5th percentile were deemed essential in transposon mutagenesis studies (20).

**TABLE 2:** Protein prediction in the 5th percentile

Category^*a*^	COG(s)	Gene(s)	Protein information
Cellular processes and signaling [D],[M],[N],[O],[T], [U],[V],[W],[Y],[Z]	D	*relB*, *relG*	Toxin-antitoxin (TA) group
	M	*rmlA*	Carbohydrate biosynthesis
	M	*ftsQ*	Essential cell division protein
	M	*mpt83*	Cell surface lipoprotein
	O	*groES*	Chaperonin GroES
	O	*ahpE* (64), *bcpB*	Peroxiredoxin (direct antioxidants)
	T	*mtrA*	Response regulator
	T	*garA*	Virulence and glutamate metabolism
	V, M	*lipP, pimA*	Role in lipid metabolism
Information storage and processing [A],[B],[J],[K],[L]	J, K, L	*tuf, trmD, greA, hupB, gatB*	Information pathways
	K	*mazE9*	Antitoxin (TA group)
	K	*whiB1, whiB2, mce3R*	Transcriptional regulator
	K	*cspB*	Cold shock protein
	K	*argR*	Amino acid biosynthesis
	K	*trcR*	Regulatory proteins
Metabolism [C],[E],[F],[G],[H],[I],[P],[Q]	C	*fdxA, fdxC*	Iron-sulfur proteins
	C	*ctaE*	Probable cytochrome oxidase
	C	*rubA*	Probable rubredoxin
	C, E	*atpA, nuoL, nuoK, pepQ*	Intermediate metabolism and respiration
	E	*metC, lysA, livN, ilvG*	Amino acid transport and metabolism
	F	*nrdI*	Ribonucleotide reductase function
	F, G, H, I	*ndkA, rpiB, nadD, thiC, dxR, gpm1*	Intermediate metabolism and respiration
	G, Q, I, P	*lppZ, lpqC, papA4, desA2*	Role in lipid metabolism
	G, P	*sugC, rfbE*	ABC transporter
	H	*dfrA, folE, folB*	Involved in folate metabolism
	H	*kdtB*	Coenzyme A (CoA) biosynthesis
	H	*mog, moaB2, moaB1, moaC2* (30)	Molybdopterin biosynthesis
	I	*mbtN*	Mycobactin biosynthesis
	P	*cysH, cysQ* (65)	Sulfate activation pathway
	P	*corA*	Transmembrane protein
Poorly characterized [R],[S]	UC	*vapB26, vapB44, vapB12, vapB9*	Antitoxin (TA group)
	S	*vapC33, vapC8, vapC7, vapC20*	Toxin (TA group)
	S	*vapC5, vapC47, vapB30, vapB32*	TA group
	S	*yfiH*	Multicopper oxidase
Unable to characterize (UC)	UC, S	*wbbL1, nicT, arfB*	Cell and cell wall-associated processes
	UC	*lsr2*	Nucleoid-associated protein Lsr2
	UC	*mazE7, mazE3*	Antitoxin (TA group)
	UC	*mymT* (37)	Metallothionein
	UC	*aprA* (66)	Acid and phagosome regulated protein
	UC	*metS*	Information pathways

a COG classification of proteins combined with information from Mycobrowser (18) and UniProt (19). Additional information from the literature, which cannot be found in these two databases, is individually cited within the table. Genes related to the metabolism of essential elements for M. tuberculosis survival, such as thiamine (e.g., *thiC*), and others related to cell envelope and active transport were also observed (e.g., *sugC*). Genes belonging to the TA family, as well as genes related to metal binding and antioxidant activity, are present in the 5th percentile. The majority of the TA genes are poorly characterized by COGs. The classification-involved proteins were encoded by genetically characterized genes with reference to H37Rv. Protein prediction for the noncharacterized genes can be found in Data Set S1 in the supplemental material.

A large number of genes that demonstrated low variation were members of the toxin-antitoxin (TA) group of genes. Of the 15 TA genes in the 5th percentile, seven encoded toxins (e.g., *vapC33*) and 10 encoded antitoxins (e.g., *vapB26*). Most of the TA genes with low variation belong to the group II TA system (e.g., *relB*, *mazE9*) (Table 2). TA genes in M. tuberculosis have been associated with many processes, including persistence and upregulation under stress conditions (29). Several highly conserved genes were related to redox processes, which can contribute to the protection of M. tuberculosis in a hostile environment (e.g., intracellular) (30). Within the 5th percentile, we observed genes encoding metal binding proteins, such as iron-sulfur proteins (*fdxa*, *fdxc*, *whiB1*, *whiB2*) and others encoding proteins that play a role in copper, zinc, or molybdenum metabolism (e.g., *moaB1*, *moaB2*, *mog*, *mymT*) (Table 2). Genes which also demonstrate a redox function or are involved in direct detoxification of peroxides and adaptation of M. tuberculosis inside the phagosome are also present in this lower part of the SNP distribution. Examples of these groups of genes are those encoding peroxiredoxins (*ahpE*, *bcpB*), rubredoxins (*rubA*), or proteins associated with the sulfur activation pathway (*cysQ*, *cysH*). Many of the genes mentioned above have been previously found to be upregulated or downregulated under stress conditions in order to possibly protect the bacillus from a hostile environment (e.g., inside macrophage) (30). We found 34 genes that have been previously identified to be influenced under stress (Table S5). Only one gene (*dfrA*) associated with drug resistance to isoniazid and *para*-aminosalicylic acid (PAS) (6, 31) was present in the 5th percentile, but its role as a resistance target has been previously debated (31).

10.1128/mSphere.01224-20.8 TABLE S5 Fifth percentile genes previously found to be up- or downregulated under stress conditions. *^a^*Thirty-four genes which have been previously identified to be influenced under stress were identified in the 5th percentile (i.e., low-variation genes), 15 of which were toxin-antitoxin (TA) genes*^b^* (1, 12, 13). *^c^*Various genes from other functional categories, such as genes associated with redox processes or adaptation within the macrophage, were also identified in the 5th percentile. These genes have been previously identified to be influenced under different stress conditions (e.g., hypoxia, nitric oxide, nutrient starvation) that resemble the hostile environment inside the macrophage during infection (1–11). Download TABLE S5, PDF file, 0.3 MB. Copyright © 2021 Papakonstantinou et al. 2021 Papakonstantinou et al. https://creativecommons.org/licenses/by/4.0/ This content is distributed under the terms of the Creative Commons Attribution 4.0 International license.

## DISCUSSION

Large-scale genomic studies have revolutionized our knowledge of M. tuberculosis drug resistance, population structure, and outbreak transmission patterns (2, 6, 11, 12). At the same time, multiple new antigens have been identified as novel drug and vaccine targets by numerous methods, including reverse vaccinology (13). A deeper understanding of the sequence variation present across all M. tuberculosis genes (and their resultant products) on a large scale could identify biologically important genes which are highly conserved. These, in turn, could be used as potential targets for novel vaccines or therapeutics. Thus, it is important to examine the levels of sequence variation in target genes, as this can directly relate to drug resistance or vaccine escape. We have combined functional annotation from multiple sources into a large-scale genomic study that provides a “snapshot” of the single-nucleotide variation of every M. tuberculosis gene and that could be used as a tool to identify desirable novel vaccine candidates and antituberculous drugs.

From our analysis, we have observed that the genes with the highest levels of single-nucleotide variation (95th percentile) included many associated with drug resistance (e.g., *gyrA*, *gidB*), pathogenesis (e.g., important gene families such as ESAT-6/ESX), and cell wall biology (e.g., *tatB*) (16, 22, 25). For example, ESAT-6 is a family of 23 proteins secreted by the ESX-1 to ESX-5 secretory proteins in pathogenic M. tuberculosis strains. Their potential role in virulence and pathogenesis and their immunogenic nature have made them promising vaccine candidates. In our study, we observed some members of the ESAT-6 and ESX-1 family with high sequence variation (95th percentile). However, any conclusions regarding these genes’ variation should be examined with caution due to the known issues of short read mapping in these repeat-rich genes (16, 32).

It is striking that a high number of genes observed in the 95th percentile have been previously identified as drug targets (22). For example, the *fadE* and *fadD* families, as well as genes such as *tatB* and *pitA*, have been previously proposed as desirable drug targets due to their functional properties and location within the organism (17, 22). *fadE* genes are associated with cholesterol metabolism, whereas *fadD* genes are involved in fatty acid metabolism. Both families are known to play a role in important biological processes, including virulence and disease pathogenesis (17, 33, 34). Similarly, genes such as *tatB* and *pitA* are both membrane transporters that have attracted the interest of the TB community as potential drug targets (35). Given the high variation of these genes in our distribution (i.e., 95th percentile), the choice of these gene products as potential drug targets should be considered with caution, as even a few single-nucleotide substitutions can have a significant impact on the binding of a drug (6). For example, if a protein possesses the desirable criteria as a drug target but its gene demonstrates high variation, perhaps several other parameters should be taken into consideration, such as the position of this variation, whether this position is in a function-critical site (e.g., active binding site), and the nature of the SNPs affected (synonymous versus nonsynonymous). Considering these points, it is important to minimize the possibility that large swaths of the M. tuberculosis population could easily become resistant to any targeting agent.

Similarly, in the 95th percentile, we have also observed genes encoding immunogenic proteins and membrane proteins with high variation that have been identified as potential vaccine candidates (e.g., *mpt53*) (21). A protein that is well recognized by adaptive immunity may not constitute a desirable vaccine target due to epitope variation in the bacterial population. If such a protein is part of a vaccine, then SNPs within the gene encoding this protein may influence the level of protection afforded after immunization (8). Moreover, this nucleotide variation may be reflected differently for targets of T-cell responses, where epitopes are linear peptides, than for targets of B-cell responses, where most epitopes are discontinuous and formed by the spatial arrangement of different regions of a protein. Nevertheless, a protein can contain multiple epitopes, and therefore SNPs may affect some responses more than others, as was recently shown for *Salmonella* (8). While it has been shown that T-cell epitopes in M. tuberculosis are generally conserved, there are exceptions to this (36). Therefore, assessing the sequence variation of each gene and the genomic positions where this variation most frequently occurs may inform future vaccine design.

Our study can be used as an overall assessment of the SNPs of any M. tuberculosis gene across a large data set. More importantly, this study provides a list of more than 80 genetically characterized genes with very low nucleotide variation in the sequenced population, the majority of which are associated with latent TB infection, survival in stress environments, and maintenance of redox balance. These genes are conserved across seven lineages, which suggests an essential role in the TB infection, and may constitute novel targets for TB drug and vaccine development. In this group of highly conserved genes (5th percentile), there are characteristic examples with interesting biological function, such as genes involved in the sulfur assimilation pathway and others associated with metal binding and metal metabolism (30, 37). Overall, these genes play a role in the maintenance of redox balance during M. tuberculosis infection (30, 38). For instance, we have observed two 7Fe ferredoxin genes (*fdxC* and *fdxA*) (39) with very low sequence variation and interesting biological function that may constitute interesting drug or vaccine targets. FdxA is a ferredoxin that has been found to be expressed under hypoxic and acidic conditions, resembling the hostile environment inside the macrophage during infection (39). FdxA is also part of the DosR regulon, a group of proteins that play a critical role during anaerobic metabolism and latent TB (40, 41). Notably, the DosR regulon is involved in latent infection, when the bacillus needs “protection” from the hostile environment inside the macrophage, and its proteins are activated by hypoxia, nitric oxide (NO), and carbon monoxide (CO) (42, 43). The DosR regulon has attracted significant interest, with vaccine candidates based on DosR proteins currently under evaluation (42, 43). Similarly, *fdxC*, which encodes another 7Fe ferredoxin, demonstrated the lowest sequence variation across our data set and has been identified as an essential gene in transposon libraries (20, 39). FdxC has not been previously synthesized; however, the crystal structure of FdxA (which shares great genetic similarity with FdxC) has already been established and can be used as a model for future FdxC synthesis (39). Redox metabolic pathways and maintenance of redox homeostasis have already been the center of interest in the development of novel M. tuberculosis drug targets. Other genes demonstrating low variation (i.e., 5th percentile) that are involved in redox homeostasis or adaptation within the macrophage are genes related to molybdenum biosynthesis (e.g., *moaB1* and *moaB2*) and others involved in sulfur metabolism (such as *cysQ*). The molybdenum metabolism plays a very important role in nitrate respiration and the ability of M. tuberculosis to persist in lung granulomas under hypoxic stress conditions (30, 44). Sulfur metabolites are part of the sulfur assimilation pathway and are essential for the M. tuberculosis adaptation and survival inside the macrophage. Key genes of the sulfur assimilation pathway seem to be induced under differential stress conditions which resemble the latent stage of TB infection. For example, CysQ is a 3′-phosphoadenosine-5′-phosphatase with an important regulatory role in M. tuberculosis sulfur pathway and has been already expressed in an Escherichia coli vector and induced under nutrient starvation (45). The conservation of some of these genes, their interesting biological properties, and the fact that they are often missing a human equivalent make them attractive drug candidates.

There are certain limitations of this study. We used H37Rv as a reference, which is a common strain used for genomic studies. H37Rv is a lineage 4 strain, known to have unique sequence variation in comparison to other strains (26, 46). We have accounted for this bias by masking all the genomic positions where more than 8,050 strains (95% of the data set) contained an identical nucleotide variant relative to H37Rv. To confirm that this masking did not lead us to miss variations present in masked sites within the population, we looked at the allelic profile of every H37Rv coordinate in our data set. The total number of multiallelic sites in our data set represents only 0.19% of the H37Rv genome (i.e., 8,220 sites), the vast majority of which are minor frequency events (median number of isolates with a variant at a multiallelic site, 3). Only 18 multiallelic sites (∼0.000408% of the entire genome) have major frequency events (i.e., a nucleotide that is present in the majority of the data set). We also acknowledge that there is differential gene content between other lineages and H37Rv (26, 47). However, the aim of this study is to specifically identify genes core to all M. tuberculosis lineages that can be used as common drug targets or vaccine candidates. In this study, we have used a large number of published genomes, which gives us a cross-representation of susceptible and resistant strains, i.e., what is currently circulating in TB. We acknowledge that we have utilized a number of drug resistance studies (see Table S1 in the supplemental material), as they are readily available online. This is probably a reflection of the great interest of the TB community in drug resistance during the last decade (6, 12, 24, 48). While the aim of this study was not to define drug resistance mutations, we show that genomic positions previously associated with drug resistance demonstrate high variation in our data set (e.g., positions 7585 and 9304 in *gyrA* or positions 4407588 and 4407927 in *gidB*) (24, 49, 50). Finally, although we appreciate the important role of mobile elements or PE-PPE genes in the M. tuberculosis genome, we have excluded them from our final analysis (along with other repetitive regions) due to the increased chance of false-positive SNPs in these regions. For instance, the PE-PPE family of genes are responsible for more than 10% of the coding capacity in the M. tuberculosis genome and are considered to play an important role in pathogenesis, antigenic variation, virulence, and immune modulation (51–53). There is also increasing interest in the scientific community in PE-PPE genes as novel drug and vaccine candidates. However, PE-PPE genes are typically excluded from SNP calling and phylogenetic reconstruction because of false-positive variants during mapping, derived from issues of mapping short-read sequencing against genes with multiple repeat regions (26, 28, 54).

In summary, we present a gene-by-gene analysis of single-nucleotide variation across a well-curated selection of 8,535 M. tuberculosis genomes as a resource to support future drug and vaccine development. We identified a number of highly conserved genes which could potentially be considered targets for novel vaccine candidates and for antituberculous medications.

## MATERIALS AND METHODS

### Sequence data and initial filtering.

We assembled a collection of genomes by utilizing large-scale genome data sets from a number of key M. tuberculosis genomic projects (see Table S1 in the supplemental material). Sequence data of 8,931 M. tuberculosis clinical isolates were downloaded from the public domain (https://www.ebi.ac.uk/ena) in the form of paired-end fastq files. The project accession numbers are available in Table S1. Reads were trimmed using Sickle (v1.33) (55) configured to a quality threshold of 20 over a 50-bp sliding window. The average sequencing depth of all genomes was determined. The final data set has a mean coverage of 103.5 with a standard deviation of 73.4.

### Variant calling and mapping.

The filtered reads were processed with Snippy (v3.2 Dev) (56) and were mapped against the reference genome for M. tuberculosis H37Rv (54). Snippy was configured to require a minimum base quality of 20, a minimum read coverage of 10, and a minimum allele frequency of 0.9 in order to obtain high-confidence variants. Snippy-core was used to determine the mapping coverage of each sequence, and a cutoff of 90% mapping coverage was chosen to filter the data set. This specific cutoff was selected after mapping three Mycobacterium canettii strains (which shares a common ancestor with M. tuberculosis) against H37Rv and determining the mapping coverage. All sequences below 90% mapping coverage were discarded. Following the application of all the quality control measures mentioned in the above sections, the data set contained a net total of 8,535 sequences.

The output from Snippy was concatenated and manually processed in order to identify variants on both a per-sample and a data set-wide basis. Further processing was performed using a script in R (https://github.com/Danaipap/TB_project). The tab-delimited output files derived from each distinct genome’s Snippy analysis (i.e., snps.tab) were combined and processed in R in order to produce a single file, which contained information regarding global genome positions (positions 1 to 4411532, as defined in H37Rv), the total number of variants at those positions, and the corresponding gene and protein names. The H37Rv reference genome contains multiple overlapping areas within coding positions. When a variant occurs in one of these areas, Snippy v3.2 Dev (using SNPef) reports the mutation in only one of the coding regions, not the other. This could potentially result in underestimating the number of variants in some overlapping genomic areas. Using the bedtools (57) intersect function, we “intersected” the reference GenBank file with a file containing the exact number of H37Rv genomic positions and the genome ID. Subsequently, we combined this intersected reference file with our combined output file. We excluded noncoding regions, insertions-deletions (indels), and RNAs from our subsequent analysis, as the focus of this study was to assess the overall nucleotide variation in genes core to all M. tuberculosis lineages that can be used as common drug targets or vaccine candidates. We also excluded mobile elements, repeat regions, transposases, and all the PE-PPE family genes from the subsequent analysis, accounting for the known caveat of false-positive SNPs in these genes during mapping (26, 28).

To account for the differential presence or absence of any given gene in the data set, we included only genes that were present at a depth of 10 and greater (minimum required for mapping) across 95% of all tested isolates. To account for the possibility that many variants are unique to H37Rv and so appear with high variation in our analysis (i.e., all genomes are identical but different from the H37Rv genome), we masked all positions where more than 8,050 strains (95% of the data set) contained an identical nucleotide variant relative to H37Rv. This extra validation was performed since H37Rv is a lineage 4 strain and it is known that it carries a lot of sequence variation not present in any other M. tuberculosis strain (26). A file describing variants at each nucleotide position in H37Rv (prior to masking and exclusion of certain sites such as those for PE-PPE, etc.) is available at https://github.com/Danaipap/TB_project.

### Determining a statistical threshold for areas with higher and lower variability than normal.

We normalized our results by dividing the number of mutations in a given gene by the gene’s length. It was noted that the distribution of genes with single-nucleotide polymorphisms (SNPs) across the 8,535 mapped genome sequences was not normal. We introduced a systematic way to analytically quantify “low” and “high” levels of sequenced variation. We defined this notion through the rate of change according to which the distribution increases and chose a cutoff where the distribution becomes stable.

We looked at the sequence of the values in each percentile. We call this sequence *f* <*f*_0_ = 0.004553734, *f*_1_ = 0.085642255… *f*_100_ = 55.166666667> (see Data Set S2 in the supplemental material). We used the ratio ρ*_i_* = *f_i_*_+ 1_/*f_i_* to determine the cutoff point where stabilization occurs in the sequence. Since the ratio is >1, the discrete sequence *f* fits as a mild exponential function. We determined the low-variability segment of the sequence as the points where the function has stabilized to attain the approximate form of *c^n^*, for a constant *c*. To achieve this, we devised a method that is governed by two parameters: (i) δ, which is the allowed fluctuation |ρ*_i_*_+ 1_ − ρ*_i_*| ≤ δ, and (ii) L, the window length (consecutive values) inside which the fluctuation is at most δ. We chose a δ of 0.03 and an *L* of 3. That is, we looked to satisfy |ρ*_i_*_+ 1_ − ρ*_i_*| ≤ 0.03 for three consecutive values. For this choice of parameters, we identified as the first stabilization point 5% of the distribution (mutations/bp). Therefore, we chose this position where the stabilization occurs to be our cutoff for this study. Symmetrically, we chose the other side of the distribution (95%), which is typical for statistical studies.

10.1128/mSphere.01224-20.10 DATA SET S2 A list of every M. tuberculosis gene, mapped against H37Rv, and its nucleotide variation across a data set of 8,535 M. tuberculosis genome sequences. Download Data Set S2, XLSX file, 0.3 MB. Copyright © 2021 Papakonstantinou et al. 2021 Papakonstantinou et al. https://creativecommons.org/licenses/by/4.0/ This content is distributed under the terms of the Creative Commons Attribution 4.0 International license.

### Phylogenetic and population structure analysis.

We used raxml with rapid bootstrapping (58) (100 bootstrap replicates) to reconstruct a core SNP phylogeny, using a general GTR+CAT approximation algorithm. The likelihood of the final tree was evaluated and optimized under GAMMA. The multifasta alignment of 8,535 genome sequences was created using the snippy-core function in Snippy. All the PE-PPE regions were masked, and the produced alignment was subsequently cleaned with the snippy-clean function. A core SNP alignment was extracted using the program snp-sites (v2.4.0) (59). One *M. canettii* genome was used as an outgroup. The phylogeny was visualized and processed further in Itol (v3) (60). Additional population structure analysis was performed with fastBAPS (61) (v1.0.0) (hierarchical Bayesian statistical clustering) to determine specific clusters within our sequenced data and to aid with the annotation of the phylogenetic tree at https://github.com/Danaipap/TB_project.

### Functional annotation and comparison of group of genes in the 5th and 95th percentiles.

Functional annotation of loci in the 5th and 95th percentiles of the diversity distribution was performed with EggNOG-mapper (v4.5) (62), and clusters of orthologous protein groups (COGs) were assigned. Individual loci were extracted using GBKsplit (63) (https://github.com/stevenjdunn/gbkSPLIT). UniProt (19) and Mycobrowser (18) databases were used in order to complement the functional annotation of the proteins. A chi-square test with Yates’ continuity correction for the comparison of the frequency of gene groups in the 5th and 95th percentiles was performed in R, and a *P* value of <0.05 was deemed significant.

### Data availability.

Accession numbers for the reads used in this project along with the information regarding the year and place of isolation are listed in Table S1 in the supplemental material.
