# Supplementary figures and images for: Mapping Gene-by-Gene Single-Nucleotide Variation in 8,535 Mycobacterium tuberculosis Genomes: a Resource To Support Potential Vaccine and Drug Development
Source: mSphere. 2021 Mar 10;6(2):e01224-20. doi: 10.1128/mSphere.01224-20 (PMC8546714; doi:10.1128/mSphere.01224-20)

Tree scale:

**Colored ranges**

- Lineage 1
- Lineage 2
- Lineage 3
- Lineage 4
- Lineage 5
- Lineage 6
- Lineage 7

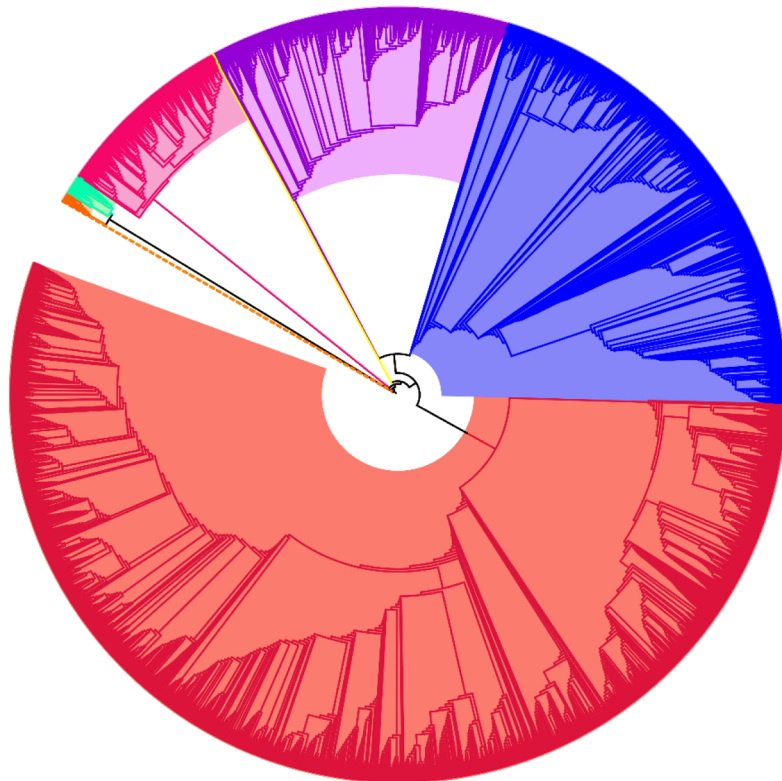

Supplement: FIG S1 [file msphere.01224-20-sf001.pdf]

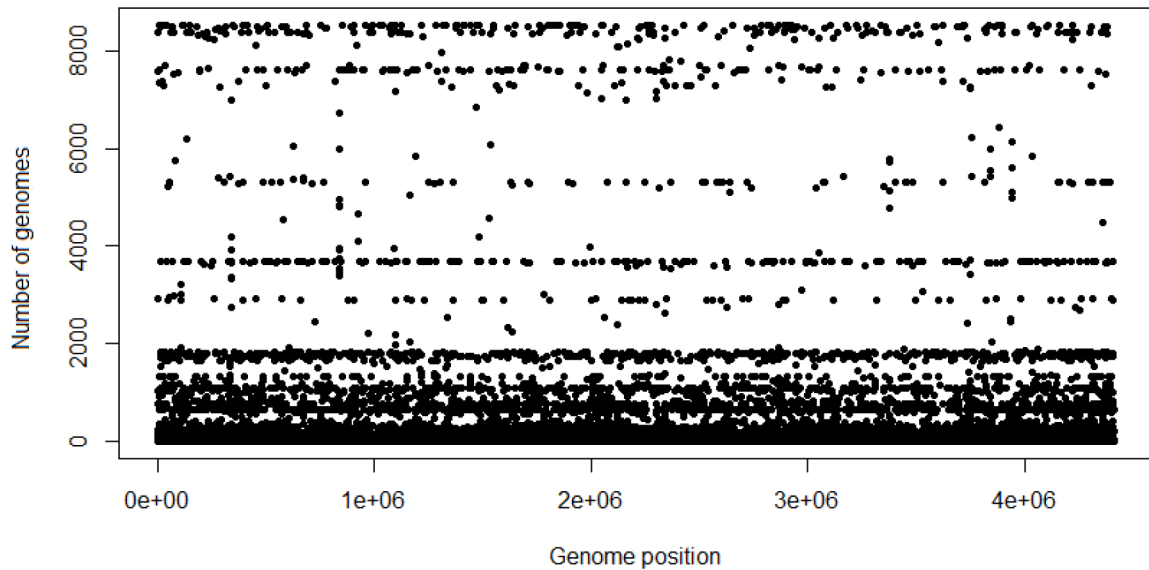

Supplement: FIG S2 [file msphere.01224-20-sf002.pdf]

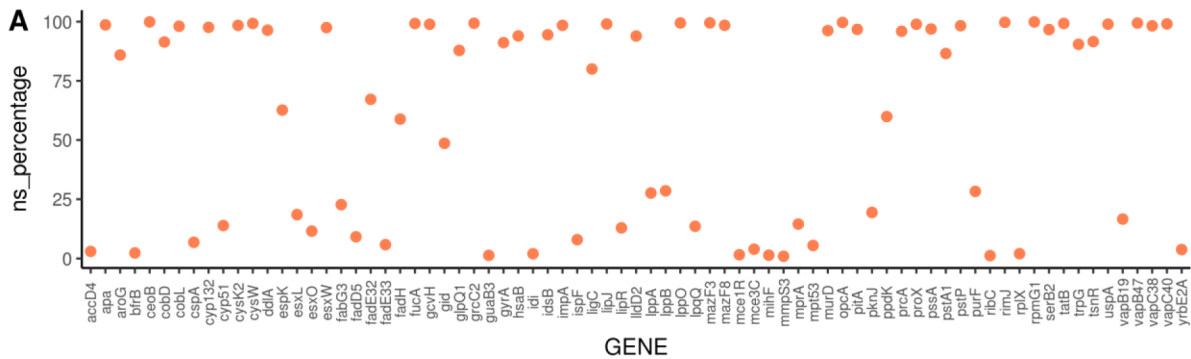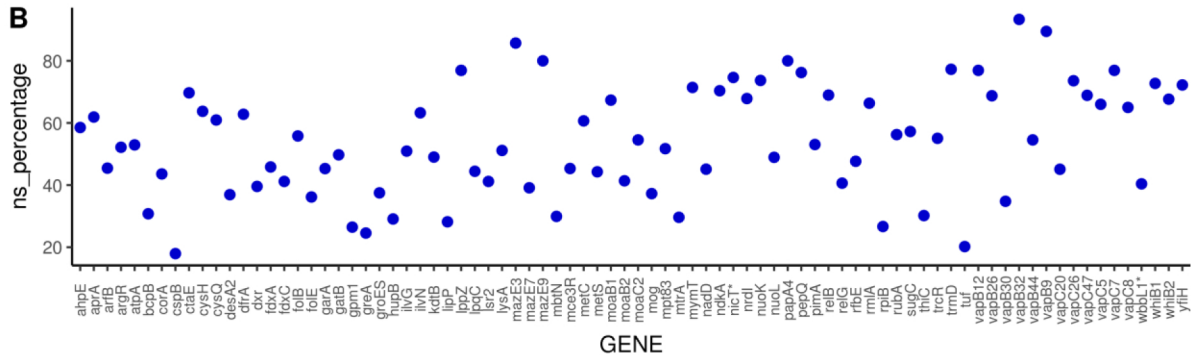

Supplement: FIG S3 [file msphere.01224-20-sf003.pdf]
