# Supplementary material for: Mapping Gene-by-Gene Single-Nucleotide Variation in 8,535 Mycobacterium tuberculosis Genomes: a Resource To Support Potential Vaccine and Drug Development
Source: mSphere. 2021 Mar 10;6(2):e01224-20. doi: 10.1128/mSphere.01224-20 (PMC8546714; doi:10.1128/mSphere.01224-20)
Supplement: TABLE S1 [file msphere.01224-20-st001.pdf]

Table S1. Datasets included in the analysis[a].

Year	Study	Accession	Countries
**2012**	Blouin *et al*, *PLoS One* (1)	ERP001885	Republic of Djibouti, Somalia, Sudan, Kenya
**2013**	Bryant *et al*,*BMC* (2)	ERP000111	Netherlands
**2013**	Clark *et al*,*PLoS One* (3)	ERP000520	Uganda
**2013**	Comas *et al, Nat. Genet* (4)	ERP001731	UK, Germany, USA, Africa(multiple countries), S. Asia, S.E. Asia, India, Uzbekistan, Netherlands Switzerland, Russia, Nepal, China, S. America
**2013**	Roetzer *et al*, *PLoS One* (5)	ERP006619	Germany
**2013**	Zhang *et al, Nat. Genet* (6)	SRP018402	China
**2014**	Casali *et al*, *Nat. Genet* (7)	ERP000192	Russia
**2014**	Perdigao *et al*, *BMC Genomics* (8)	ERP002611	Portugal
**2015**	Ali *et al, PLoS One* (9)	PRJEB7798	Pakistan
**2015**	Assuncao *et al*, *e –Life* (10)	ERP000436, ERP001072,	Malawi
**2015**	Merker *et al, Nat. Genet* (11)	PRJEB7281	Eurasia
**2015**	Walker *et al, Lancet ID* (12)	PRJNA282721	UK, Sierra Leone, S Africa, Germany, Uzbekistan
**2015**	Lee *et al*, *PNAS* (13)	SRP039605	Inuit
**2016**	Guimaraes *et al, Microb Genom* (14)	PRJEB8432	Ethiopia
**2016**	Stucki *et al, Nat. Genet* (15)	PRJEB11460	Africa, Nepal, Papua New Guinea, Switzerland
**2017**	Chatterjee *et al,Tuberculosis* (16)	SRP101835	India
**2018**	Chernyaeva *et al, EID* (17)	PRJNA352769	Russia

References

1. Blouin Y, Hauck Y, Soler C, Fabre M, Vong R, Dehan C, Cazajous G, Massoure P-L, Kraemer P, Jenkins A, Garnotel E, Pourcel C, Vergnaud G. 2012. Significance of the Identification in the Horn of Africa of an Exceptionally Deep Branching Mycobacterium tuberculosis Clade. PLoS One 7.

2. Bryant JM, Schürch AC, van Deutekom H, Harris SR, de Beer JL, de Jager V, Kremer K, van Hijum SAFT, Siezen RJ, Borgdorff M, Bentley SD, Parkhill J, van Soolingen D. 2013. Inferring patient to patient transmission of Mycobacterium tuberculosisfrom whole genome sequencing data. BMC Infect Dis 13:110.

3. Clark TG, Mallard K, Coll F, Preston M, Assefa S, Harris D, Ogwang S, Mumbowa F, Kirenga B, O 'sullivan DM, Okwera A, Eisenach KD, Joloba M, Bentley SD, Ellner JJ, Parkhill J, Jones-López EC, Mcnerney R. 2013. Elucidating Emergence and Transmission of Multidrug- Resistant Tuberculosis in Treatment Experienced Patients by Whole Genome Sequencing. PLOS ONE | www.plosone.org 1 8:e:83012.

4. Comas I, Coscolla M, Luo T, Borrell S, Holt KE, Kato-Maeda M, Parkhill J, Malla B, Berg S, Thwaites G, Yeboah-Manu D, Bothamley G, Mei J, Wei L, Bentley S, Harris SR, Niemann S, Diel R, Aseffa A, Gao Q, Young D, Gagneux S. 2013. Out-of-Africa migration and Neolithic co-expansion of Mycobacterium tuberculosis with modern humans. Nat Genet 45:1176–1182.

5. Roetzer A, Diel R, Kohl TA, Rückert C, Nübel U, Blom J, Wirth T, Jaenicke S,

Schuback S, Rüsch-Gerdes S, Supply P, Kalinowski J, Niemann S. 2013. Whole Genome Sequencing versus Traditional Genotyping for Investigation of a Mycobacterium tuberculosis Outbreak: A Longitudinal Molecular Epidemiological Study. PLoS Med 10:e1001387.

6. Zhang H, Li D, Zhao L, Fleming J, Lin N, Wang T, Liu Z, Li C, Galwey N, Deng J, Zhou Y, Zhu Y, Gao Y, Wang T, Wang S, Huang Y, Wang M, Zhong Q, Zhou L, Chen T, Zhou J, Yang R, Zhu G, Hang H, Zhang J, Li F, Wan K, Wang J, Zhang X-E, Bi L. 2013. Genome sequencing of 161 Mycobacterium tuberculosis isolates from China identifies genes and intergenic regions associated with drug resistance. Nat Genet 45:1255–1260.

7. Casali N, Nikolayevskyy V, Balabanova Y, Harris SR, Ignatyeva O, Kontsevaya I, Corander J, Bryant J, Parkhill J, Nejentsev S, Horstmann RD, Brown T, Drobniewski F. 2014. Evolution and transmission of drug resistant tuberculosis in a Russian population Europe PMC Funders Group. Nat Genet 46:279–286.

8. Perdigão J, Silva H, Machado D, Macedo R, Maltez F, Silva C, Jordao L, Couto I, Mallard K, Coll F, Hill-Cawthorne GA, Mcnerney R, Pain A, Clark TG, Viveiros M, Portugal I. 2014. Unraveling Mycobacterium tuberculosis genomic diversity and evolution in Lisbon, Portugal, a highly drug resistant setting. BMC Genomics 15:1–20.

9. Ali A, Hasan Z, McNerney R, Mallard K, Hill-Cawthorne G, Coll F, Nair M, Pain A, Clark TG, Hasan R. 2015. Whole Genome Sequencing Based

Characterization of Extensively Drug-Resistant Mycobacterium tuberculosis Isolates from Pakistan. PLoS One 10:e0117771.

10. Guerra-Assunção JA, Crampin AC, Houben RMGJ, Mzembe T, Mallard K, Coll F, Khan P, Banda L, Chiwaya A, Pereira RPA, McNerney R, Fine PEM, Parkhill J, Clark TG, Glynn JR. 2015. Large-scale whole genome sequencing of M. tuberculosis provides insights into transmission in a high prevalence area. Elife 4:1–17.

11. Merker M, Blin C, Mona S, Duforet-Frebourg N, Lecher S, Willery E, Blum MGB, Rüsch-Gerdes S, Mokrousov I, Aleksic E, Allix-Béguec C, Antierens A, Augustynowicz-Kopeć E, Ballif M, Barletta F, Beck HP, Barry CE, Bonnet M, Borroni E, Campos-Herrero I, Cirillo D, Cox H, Crowe S, Crudu V, Diel R, Drobniewski F, Fauville-Dufaux M, Gagneux S, Ghebremichael S, Hanekom M, Hoffner S, Jiao WW, Kalon S, Kohl TA, Kontsevaya I, Lillebæk T, Maeda S, Nikolayevskyy V, Rasmussen M, Rastogi N, Samper S, Sanchez-Padilla E, Savic B, Shamputa IC, Shen A, Sng LH, Stakenas P, Toit K, Varaine F, Vukovic D, Wahl C, Warren R, Supply P, Niemann S, Wirth T. 2015. Evolutionary history and global spread of the Mycobacterium tuberculosis Beijing lineage. Nat Genet 47:242–249.

12. Walker TM, Kohl TA, Omar S V, Hedge J, Del Ojo Elias C, Bradley P, Iqbal Z, Feuerriegel S, Niehaus KE, Wilson DJ, Clifton DA, Kapatai G, C Ip CL, Bowden R, Drobniewski FA, Allix-Béguec C, Gaudin C, Parkhill J, Diel R, Supply P, Crook DW, Grace Smith E, Sarah Walker A, Ismail N, Niemann S, A Peto TE. 2015. Whole-genome sequencing for prediction of Mycobacterium

tuberculosis drug susceptibility and resistance: a retrospective cohort study. www.thelancet.com/infection 15:1193–1202.

13. Lee RS, Radomski N, Proulx J-F, Levade I, Shapiro BJ, Mcintosh F, Soualhine H, Menzies D, Behr MA, Nathan CF. 2015. Population genomics of Mycobacterium tuberculosis in the Inuit. PNAS 112:13609–13614.

14. Nebenzahl-Guimaraes H, Yimer SA, Holm-Hansen C, De Beer J, Brosch R, Van Soolingen D. 2016. Genomic characterization of Mycobacterium tuberculosis lineage 7 and a proposed name: "Aethiops vetus." Microb genomics 1–8.

15. Stucki D, Brites D, Jeljeli L, Coscolla M, Liu Q, Trauner A, Fenner L, Rutaihwa L, Borrell S, Luo T, Gao Q, Kato-Maeda M, Ballif M, Egger M, Macedo R, Mardassi H, Moreno M, Vilanova GT, Fyfe J, Globan M, Thomas J, Jamieson F, Guthrie JL, Asante-Poku A, Yeboah-Manu D, Wampande E, Ssengooba W, Joloba M, Boom WH, Basu I, Bower J, Saraiva M, Vasconcellos SEG, Suffys P, Koch A, Wilkinson R, Gail-Bekker L, Malla B, Ley SD, Beck HP, De Jong BC, Toit K, Sanchez-Padilla E, Bonnet M, Gil-Brusola A, Frank M, Penlap Beng VN, Eisenach K, Alani I, Ndung'U PW, Revathi G, Gehre F, Akter S, Ntoumi F, Stewart-Isherwood L, Ntinginya NE, Rachow A, Hoelscher M, Cirillo DM, Skenders G, Hoffner S, Bakonyte D, Stakenas P, Diel R, Crudu V, Moldovan O, Al-Hajoj S, Otero L, Barletta F, Carter EJ, Diero L, Supply P, Comas I, Niemann S, Gagneux S. 2016. Mycobacterium tuberculosis lineage 4 comprises globally distributed and geographically restricted sublineages. Nat Genet 48:1535–1543.

16. Chatterjee A, Nilgiriwala K, Saranath D, Rodrigues C, Mistry N. 2017. Whole genome sequencing of clinical strains of Mycobacterium tuberculosis from Mumbai, India: A potential tool for determining drug-resistance and strain lineage. Tuberculosis 107:63–72.

17. Chernyaeva E, Rotkevich M, Krasheninnikova K, Yurchenko A, Vyazovaya A, Mokrousov I, Solovieva N, Zhuravlev V, Yablonsky P, O’Brien SJ. 2018. Whole-Genome Analysis of Mycobacterium tuberculosis from Patients with Tuberculous Spondylitis, Russia. Emerg Infect Dis 24:579–583.
