# Supplementary material for: Mapping Gene-by-Gene Single-Nucleotide Variation in 8,535 Mycobacterium tuberculosis Genomes: a Resource To Support Potential Vaccine and Drug Development
Source: mSphere. 2021 Mar 10;6(2):e01224-20. doi: 10.1128/mSphere.01224-20 (PMC8546714; doi:10.1128/mSphere.01224-20)
Supplement: TABLE S2 [file msphere.01224-20-st002.pdf]

Table S2.

**TUBERCULIST-95th**	**95th (%)**	**COGs[b]-95th**	**95th (%)**
Cell wall and cell processes [a]	24.2 %	Poorly characterised	39.2%
Hypotheticals & Unknown	33.9 %	Metabolic proteins	34.4%
Information pathways	4.3 %	Information storage	15.1%
Intermediary metabolism & respiration	19.4 %	Cellular processes & signalling	11.3%
Lipid metabolism	4.3 %		
Regulatory proteins	4.84 %		
Virulence, detoxification, adaptation	6.45 %		
Insertion seqs & phages	2.69 %		
**TUBERCULIST-5th**	**5th (%)**	**COGs[b]-5th**	**5th (%)**
Cell wall and cell processes	11.8 %	Poorly characterised	39.8%
Hypotheticals & Unknown	34.4 %	Metabolic proteins	29%
Information pathways	7.53 %	Information storage	22.6%
Intermediary metabolism & respiration	21.5 %	Cellular processes & signalling	8.6%
Lipid Metabolism	2.15 %		
Regulatory proteins	6.45 %		
Virulence, detoxification, adaptation	12.4 %		
Insertion seqs & phages	3.76 %		
