# Supplementary material for: Mapping Gene-by-Gene Single-Nucleotide Variation in 8,535 Mycobacterium tuberculosis Genomes: a Resource To Support Potential Vaccine and Drug Development
Source: mSphere. 2021 Mar 10;6(2):e01224-20. doi: 10.1128/mSphere.01224-20 (PMC8546714; doi:10.1128/mSphere.01224-20)
Supplement: TABLE S3 [file msphere.01224-20-st003.pdf]

Table S3.

Groups of genes	Genes-5th	Genes-95th	X^{2a}	p-value [e]
**COGs categories** [b]				
Cellular processes & signalling	16	21	0.48019	0.4883
Information storage & processing	42	28	2.9739	0.08462
Poorly characterised	74	73	0	1
Metabolism	54	64	1.0053	0.316
**Tuberculist categories** [c]				
Cell & cell wall processes	22	45	8.8108	*0.002995*
Hypotheticals/unknown	64	63	0	1
Information pathways	14	8	1.2078	0.2718
Intermediate metabolism/ respiration	40	36	0.14883	0.6997
Virulence, detoxification, adaptation	23	12	3.1539	0.07575
Insertion seqs & phages	7	5	0.086111	0.7692
Lipid Metabolism	4	8	0.775	0.3787
Regulatory proteins	12	9	0.20187	0.6532
**Other categories**				
Toxin-antitoxin [d]	17	5	5.0244	*0.02499*
ESAT6/ESX	0	4	2.2745	0.1315
Resistance genes	1	7	3.1937	0.07392
Induced under stress [d]	34	9	14.932	*0.0001115*
