# Supplementary material for: Mapping Gene-by-Gene Single-Nucleotide Variation in 8,535 Mycobacterium tuberculosis Genomes: a Resource To Support Potential Vaccine and Drug Development
Source: mSphere. 2021 Mar 10;6(2):e01224-20. doi: 10.1128/mSphere.01224-20 (PMC8546714; doi:10.1128/mSphere.01224-20)
Supplement: TABLE S4 [file msphere.01224-20-st004.pdf]

Table S4.

GENE [a]	FUNCTION	STRESS	OUTCOME
cspA	Cold-shock protein	Low temperature	Upregulation (1)
bfrB	Ferritin	Hypoxia, iron-stress	Upregulation (2)
cysW	Sulfate ABC transporter	Starvation	Upregulation (3)
vapC40 [b]	Toxin (TA system)	Starvation, drugs	Upregulation (4)
vapB47 [b]	Antitoxin (TA system)	Drugs	Upregulation (4)
vapB19 [b]	Antitoxin (TA system)	Starvation	Upregulation (5)
vapC38 [b]	Toxin (TA system)	Starvation	Upregulation (5)
mazF8 [b]	Antitoxin (TA system)	Various stress	Upregulation (5)
mazF3 [b]	Toxin (TA system)	Starvation, drugs, low ph	Upregulation (4)

References:

1. D'Auria G, Esposito C, Falcigno L, Calvanese L, Iaccarino E, Ruggiero A, Pedone C, Pedone E, Berisio R. 2010. Dynamical properties of cold shock protein A from Mycobacterium tuberculosis. Biochem Biophys Res Commun 402:693–698.

2. Khare G, Nangpal P, Tyagi AK. 2017. Differential Roles of Iron Storage Proteins in Maintaining the Iron Homeostasis in Mycobacterium tuberculosis. PLoS One 12:e0169545.

3. Hatzios SK, Bertozzi CR. 2011. The regulation of sulfur metabolism in Mycobacterium tuberculosis. PLoS Pathog 7:e1002036.

4. Gupta A, Venkataraman B, Vasudevan M, Gopinath Bankar K. 2017. Coexpression network analysis of toxin-antitoxin loci in Mycobacterium tuberculosis reveals key modulators of cellular stress. Sci Rep 7:1–14.

5. Sala A, Bordes P, Genevaux P. 2014. Multiple Toxin-Antitoxin Systems in Mycobacterium tuberculosis. Toxins (Basel) 6:1002–1020.
