# Supplementary material for: Mapping Gene-by-Gene Single-Nucleotide Variation in 8,535 Mycobacterium tuberculosis Genomes: a Resource To Support Potential Vaccine and Drug Development
Source: mSphere. 2021 Mar 10;6(2):e01224-20. doi: 10.1128/mSphere.01224-20 (PMC8546714; doi:10.1128/mSphere.01224-20)
Supplement: TABLE S5 [file msphere.01224-20-st005.pdf]

Table S5

GENE [a]	FUNCTION	STRESS	OUTCOME
Fdxa [c]	ferredoxin-redox	hypoxia	Upregulation (1)
mymT [c]	metallothionein-redox	high copper, NO	Upregulation (2)
cysQ [c]	Sulfur metabolism-redox	nutrient starvation	Upregulation (3)
cysH [c]	Sulfur metabolism-redox	nutrient starvation,H2O2	Upregulation (3)
moaB1 [c]	Molybdenum metabolism-redox	hypoxia, NO	Upregulation (4)
mog [c]	Molybdenum metabolism-redox	hypoxia	Upregulation (4)
whiB1 [c]	transcriptional regulator-redox	nitric oxide	Downregulation (5)
whiB2 [c]	transcriptional regulator-redox	hypoxia	Downregulation (5)
groES [c]	heat shock protein	heat	Upregulation (6)
Tuf [c]	elongation factor	hypoxia	Upregulation (7)
cspB	cold shock protein	cold	Upregulation
ilvG [c]	acetolactate synthase	hypoxia, low ph	Upregulation (8)
ilvN [c]	acetolactate synthase	hypoxia	Downregulation (8)
aprA [c]	acid & phagosome regulated protein	low ph	Upregulated (9)
arfABC [c]	ph regulator	low ph	Transformation (10)
lsr2 [c]	transcriptional regulator	hypoxia	Upregulation (11)
relB [b]	TA - Antitoxin	low ph, drugs	Upregulation (12)
relG [b]	TA - Toxin	Nitrogen stress	Upregulation (12)
mazE9 [b]	TA - Antitoxin	low ph, drugs	Upregulation (12)
vapB26 [b]	TA - Antitoxin	nutrient starvation	Upregulation (12)
vapB44 [b]	TA - Antitoxin	nutrient starvation	Upregulation (12)
vapC5 [b]	TA - Toxin	nutrient starvation	Upregulation (13)
vapB12 [b]	TA - Antitoxin	starvation, low ph, drugs	Upregulation (12)
vapB9 [b]	TA - Antitoxin	starvation, drugs	Upregulation (12)
vapC33 [b]	TA - Toxin	drugs	Upregulation(12)
vapC8 [b]	TA - Toxin	drugs	Upregulation(12)
vapC7 [b]	TA - Toxin	starvation, low ph, drugs	Upregulation(12)
vapC26 [b]	TA - Toxin	starvation, drugs	Upregulation(12)
vapC20 [b]	TA - Toxin	drugs	Upregulation(12)
vapB30 [b]	TA - Antitoxin	starvation, low ph (4.8)-24h	Upregulation(12)
vapB32 [b]	TA - Antitoxin	starvation, low ph, drugs	Upregulation(12)
mazE7 [b]	TA - Antitoxin	starvation, drugs	Upregulation(12)
mazE3 [b]	TA - Antitoxin	starvation, low ph, drugs	Upregulation(12)
vapC47 [b]	TA - Toxin	starvation, low ph, drugs	Upregulation(12)

References

1. Ramage HR, Connolly LE, Cox JS. 2009. Comprehensive Functional Analysis of Mycobacterium tuberculosis Toxin-Antitoxin Systems: Implications for Pathogenesis, Stress Responses, and Evolution. PLoS Genet 5:e1000767.
2. Gold B, Deng H, Bryk R, Vargas D, Eliezer D, Roberts J, Jiang X, Nathan C. 2008. Identification of a Copper-Binding Metallothionein in Pathogenic Mycobacteria. Nat Chem Biol 4:609–616.
3. Hatzios SK, Bertozzi CR. 2011. The regulation of sulfur metabolism in Mycobacterium tuberculosis. PLoS Pathog 7:e1002036.
4. Levillain F, Poquet Y, Mallet L, Mazères S, Marceau M, Brosch R, Bange F-C, Supply P, Magalon A, Neyrolles O. 2017. Horizontal acquisition of a hypoxia responsive molybdenum cofactor biosynthesis pathway contributed to Mycobacterium tuberculosis pathoadaptation. PLOS Pathog 13:e1006752.
5. Alam MS, Garg SK, Agrawal P. 2009. Studies on structural and functional divergence among seven WhiB proteins of Mycobacterium tuberculosis H37Rv. FEBS J 276:76–93.
6. Aravindhan V, Christy AJ, Roy S, Ajitkumar P, Narayanan PR, Narayanan S. 2009. Mycobacterium tuberculosis groE promoter controls the expression of the bicistronic *groESL1* operon and shows differential regulation under stress conditions. FEMS Microbiol Lett 292:42–49.
7. Starck J, Kä llenius G, Marklund B-I, Andersson DI. 2004. Comparative proteome analysis of Mycobacterium tuberculosis grown under aerobic and anaerobic conditions. Microbiology 150:3821–3829.
8. Singh V, Chandra D, Srivastava BS, Srivastava R. 2011. Biochemical and transcription analysis of acetohydroxyacid synthase isoforms in

Mycobacterium tuberculosis identifies these enzymes as potential targets for drug development. Microbiology 157:29–37.

9. Abramovitch RB, Rohde KH, Hsu F-F, Russell DG. 2011. aprABC: A Mycobacterium tuberculosis complex-specific locus that modulates pH-driven adaptation to the macrophage phagosome. Mol Microbiol 80:678–694.

10. Yao Y, Barghava N, Kim J, Niederweis M, Marassi FM. 2012. Molecular structure and peptidoglycan recognition of Mycobacterium tuberculosis ArfA (Rv0899). J Mol Biol 416:208–220.

11. Bartek IL, Woolhiser LK, Baughn AD, Basaraba RJ, Jacobs WR, Lenaerts AJ, Voskuil MI, Voskuil MI. 2014. Mycobacterium tuberculosis Lsr2 is a global transcriptional regulator required for adaptation to changing oxygen levels and virulence. MBio 5:e01106-14.

12. Gupta A, Venkataraman B, Vasudevan M, Gopinath Bankar K. 2017. Co-expression network analysis of toxin-antitoxin loci in Mycobacterium tuberculosis reveals key modulators of cellular stress. Sci Rep 7:1–14.

13. Sala A, Bordes P, Genevaux P. 2014. Multiple Toxin-Antitoxin Systems in Mycobacterium tuberculosis. Toxins (Basel) 6:1002–1020.
